# Comparison of Phenotypes between Different *vangl2* Mutants Demonstrates Dominant Effects of the *Looptail* Mutation during Hair Cell Development

**DOI:** 10.1371/journal.pone.0031988

**Published:** 2012-02-20

**Authors:** Haifeng Yin, Catherine O. Copley, Lisa V. Goodrich, Michael R. Deans

**Affiliations:** 1 The Departments of Neuroscience and Otolaryngology-Head and Neck Surgery, the Center for Hearing and Balance and the Center for Sensory Biology, The Johns Hopkins University School of Medicine, Baltimore, Maryland, United States of America; 2 The Department of Neurobiology, Harvard Medical School, Boston, Maryland, United States of America; MRC, University College of London, United Kingdom

## Abstract

Experiments utilizing the *Looptail* mutant mouse, which harbors a missense mutation in the *vangl2* gene, have been essential for studies of planar polarity and linking the function of the core planar cell polarity proteins to other developmental signals. Originally described as having dominant phenotypic traits, the molecular interactions underlying the *Looptail* mutant phenotype are unclear because Vangl2 protein levels are significantly reduced or absent from mutant tissues. Here we introduce a *vangl2* knockout mouse and directly compare the severity of the knockout and *Looptail* mutant phenotypes by intercrossing the two lines and assaying the planar polarity of inner ear hair cells. Overall the *vangl2* knockout phenotype is milder than the phenotype of compound mutants carrying both the *Looptail* and *vangl2* knockout alleles. In compound mutants a greater number of hair cells are affected and changes in the orientation of individual hair cells are greater when quantified. We further demonstrate in a heterologous cell system that the protein encoded by the *Looptail* mutation (Vangl2^S464N^) disrupts delivery of Vangl1 and Vangl2 proteins to the cell surface as a result of oligomer formation between Vangl1 and Vangl2^S464N^, or Vangl2 and Vangl2^S464N^, coupled to the intracellular retention of Vangl2^S464N^. As a result, Vangl1 protein is missing from the apical cell surface of vestibular hair cells in *Looptail* mutants, but is retained at the apical cell surface of hair cells in *vangl2* knockouts. Similarly the distribution of Prickle-like2, a putative Vangl2 interacting protein, is differentially affected in the two mutant lines. In summary, we provide evidence for a direct physical interaction between Vangl1 and Vangl2 through a combination of *in vitro* and *in vivo* approaches and propose that this interaction underlies the dominant phenotypic traits associated with the *Looptail* mutation.

## Introduction

Planar polarity is the polarized organization of cells and cellular structures within the plane of a tissue, perpendicular to the apical-basolateral cell axis [1], [2], [3]. In the sensory epithelia of the vertebrate inner ear, planar polarity is evident in the organization of a stereocilia bundle and kinocilium atop the sensory receptor hair cells that mediate hearing and balance. Movements of the bundle towards the kinocilium produce excitatory electrophysiological responses in the hair cell, while movements away are inhibitory. As a result, loss of planar polarity for even a subset of auditory hair cells is sufficient to produce a measurable decrease in auditory function [4]. Individual hair cells are polarized by the asymmetric, subcellular distribution of the core Planar Cell Polarity (PCP) proteins including Frizzled (Fz), Dishevelled (Dvl), Van Gogh (Vangl), Prickle (Pk) and CELSR [3]. The polarized distribution of these proteins in vestibular hair cells of the mouse appears to be highly conserved, resembling the organization of core PCP proteins in epithelia cells of the *Drosophila* wing. Taken together, several recent studies indicate that Fz and Dvl proteins form a complex on one side of the hair cell that is opposite to Vangl and Pk proteins on the other [5], [6], [7], [8]. Similar to *Drosophila*, vertebrate PCP proteins also coordinate planar polarity between cells so that, as in the case of hair cells, the stereocilia bundles of neighboring cells are oriented in the same direction.

An early demonstration the functional significance of planar polarity and PCP signaling mechanisms in hair cells came from analysis of the *vangl2* (Entrez Gene ID: 93840) mutant line *Looptail (Lp)* [9], [10]. *Lp* is a missense mutation of *vangl2* resulting in a Serine to Asparagine substitution at amino acid position 464, which is located in the cytoplasmic domain of the protein. In *vangl2^Lp/Lp^* mutants, hair cell stereocilia are properly formed and polarized, yet individual hair cells are frequently misoriented relative to neighboring cells in the cochlea and utricle [7], [11]. A greater proportion of hair cells are misoriented in the cochlea of embryos with mutations in both *vangl1* (Entrez Gene ID: 229658) and *vangl2* demonstrating functional redundancy of the two homologous genes [12], [13]. In additional to hair cell planar polarity phenotypes, *vangl2^Lp/Lp^* mutants have craniorachischisis, which is a severe neural tube defect (NTD). The iconic looped tail of the *Lp* line is due to a milder neural tube phenotype in heterozygous mice. Craniorachischisis is a class of NTD specifically resulting from disrupted planar polarity during convergent extension of the embryo prior to neurulation, and is a common feature of vertebrate PCP mutants. Other mouse lines that develop craniorachischisis and hair cell planar polarity defects include *fz3/6* double knockouts [8], *CELSR* mutants *spin cycle* and *crash* [14], *scribble* mutants [15], and *ptk7* knockout mice [16]. In addition, mutations in *vangl1* and *vangl2* have been linked to neural tube defects in humans [17], [18].

The *Lp* mouse has served as an important animal model for studying vertebrate planar polarity [11] that is frequently used to query genetic interactions between PCP and other signaling pathways [19], [20], [21]. The original characterization of the *Lp* line classified the *Lp* mutation as having a partially penetrant and dominant phenotype [22]. Consistent with these conclusions, when female reproductive tract explants are cultured *ex vivo*, heterozygous tissue develops pathologic characteristics similar to mutant tissue [23]. A confounding factor however, is that the severity of the phenotype can also be strain dependent suggesting the additional influence of genetic modifiers [3]. Moreover, in the time since the *Lp* mutation was mapped to *vangl2* [9], [10], molecular assays have demonstrated reduced Vangl2^S464N^ protein stability and expression in *vangl2^Lp/Lp^* mutants [7], [24], [25]. This has led to a reinterpretation of *Lp* as a null allele with a heterozygous phenotype resulting from haploinsufficiency. The *Lp* line has implicated PCP signaling in a number of developmental events such as axon pathfinding not originally associated with planar polarity [24]. In addition, genetic interactions between *Lp* and other mutant lines suggest that other signaling pathways, including those functional in cilia, incorporate Vangl2 [20], [26]. In order to accurately interpret Vangl2 function in these different contexts it is important to establish the true molecular basis and phenotypic nature of the *Lp* mutation.

One explanation is that the *Lp* mutation inhibits Vangl2 function by disrupting Vangl2 protein trafficking through the endoplasmic reticulum (ER). Consistent with this hypothesis, mutations in *sec24b*, which encodes an ER transport protein that is required for Vangl2 movement to the plasma membrane, result in craniorachischisis similar to *Lp* [27], [28]. Similarly Vangl2^S464N^ is not loaded into ER vesicles indicating that the mutated region of the Vangl2 cytoplasmic domain may be required for sorting by Sec24b. Furthermore, a second *vangl2* point mutant (*m1Jus*) fails to enter ER vesicles [27], and *vangl2^m1Jus/m1Jus^* mice have craniorachischisis similar to *vangl2^Lp/Lp^* [29]. Moreover mutant Vangl2 trafficking deficits are consistent with the reduced levels of Vangl2 protein detected in mutant tissues [25] and unstable recombinant mutant protein reported *in vitro* [7]. Still it is unclear whether the heterozygous phenotype of *vangl2^Lp/WT^* mice is due to haploinsufficiency or if it is a partially penetrant, dominant phenotype as described in the original characterization of the line.

We have generated a complementary mouse line in which a single exon encoding the Vangl2 transmembrane domains is disrupted by LoxP site addition and subsequent excision by Cre recombinase. At the chromosomal level, the mutation in these *vangl2* knockout mice is more severe than the *Lp* and *m1Jus* mutations because remaining elements of *vangl2* are not sufficient to encode a membrane spanning protein. Using the sensory hair cells of the inner ear, we compared the mutant phenotype of *vangl2* knockout and *Lp* mutant mice to establish the phenotypic nature of the *Lp* mutation during hair cell development.

## Results

**Figure 1:**
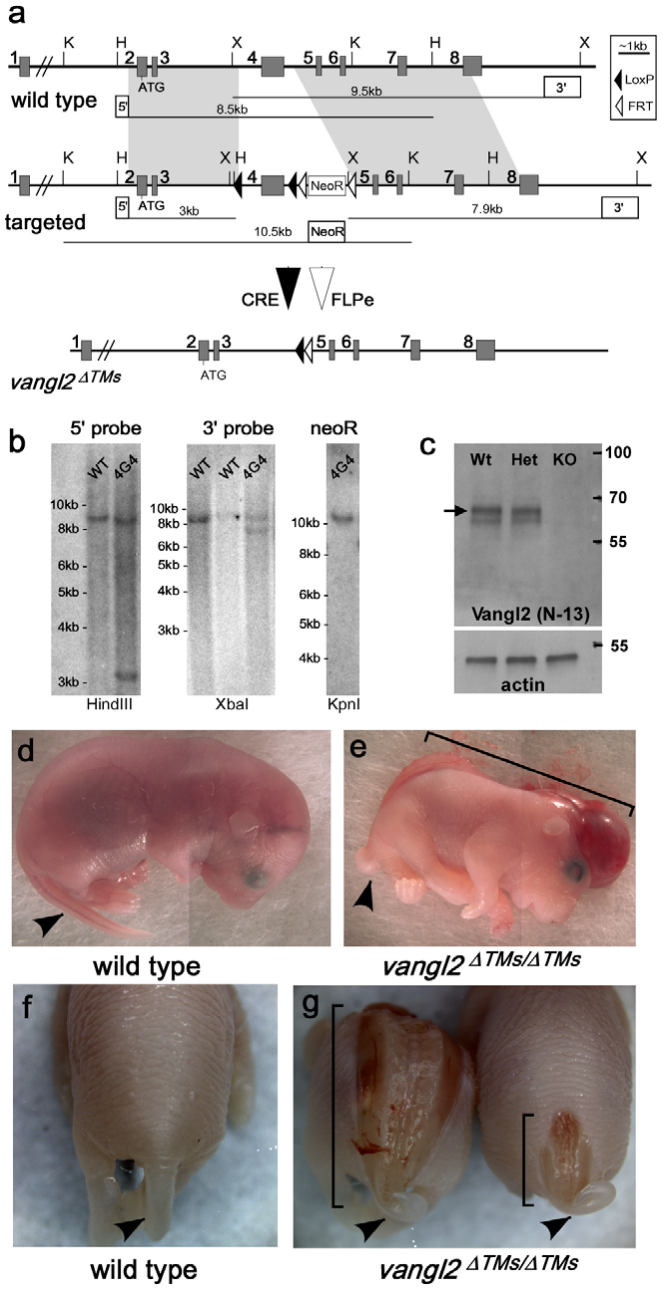
Gene targeting strategy and observations of neural tube defects in *vangl2^ΔTMs/ΔTMs^* mice. (**A**) *vangl2* gene function was disrupted by genetically modifying the wild type allele to contain two LoxP sequences flanking exon 4. A neomycin-resistance selection cassette (NeoR) with FRT sites was included for selecting recombinant clones. Regions of homologous recombination between the wild type and targeted gene are indicated by light gray shading. Following Cre and FlpE-mediated recombination events the targeted allele is reduced to the *vangl2^ΔTMs^* knockout allele. The position of KpnI (K), HindIII (H), and XbaI (X) restriction endonuclease sites and the location of probes (5′, 3′ and NeoR) used for Southern blot analysis are indicated. (**B**) Southern blot against recombinant ES cells using 5′ and 3′ probes revealed homologous integration in line 4G4 and additional blots using a NeoR probe confirmed a single insertion event. (**C**) Western blot against protein lysates from embryonic tissues demonstrate the complete loss of Vangl2 protein from *vangl2^ΔTMs/ΔTMs^* (KO) embryos. Phosphorylated Vangl2 runs in a slower migrating band (arrow) in denaturing SDS-PAGE gels resulting in a doublet. Blots for Actin were used as a loading control. (**D,E**) At E18.5 many *vangl2^ΔTMs/ΔTMs^* have craniorachischisis. (**F,G**) Representative images of the posterior tail region that demonstrate the milder spina bifida defects that occur in 26% of *vangl2^ΔTMs/ΔTMs^* embryos. Arrowheads mark the position of the tail (D–G) and brackets mark the length of the open neural tube (E,G).

To further evaluate Vangl2 function during sensory hair cell development, a *vangl2* knockout (KO) mouse was generated and compared to the established *Looptail* (*Lp*) mouse line to determine the effect of the S464N substitution on Vangl2 function. The *vangl2* gene was modified by homologous recombination in ES cells to introduce tandem LoxP sites flanking exon 4, which encodes the four Vangl2 transmembrane domains (Fig. 1A). Accurate recombination was confirmed by Southern blot assay, and a single line was used for blastocyst injections (Fig. 1B). Following germline transmission of the targeted allele, exon 4 and the NeoR selection cassette were permanently deleted by sequentially crossing the line with transgenic mice expressing ubiquitous Cre recombinase [30] or FLPe [31] (Fig. 1A). Since targeting exon 4 deletes the Vangl2 transmembrane domains, this knockout allele is called *vangl2^ΔTMs^* to distinguish it from other *vangl2* alleles. Western blot analyses of whole brain lysates using an antibody against the amino terminus of Vangl2 (N-13) confirms a loss of Vangl2 protein in *vangl2^ΔTMs/ΔTMs^* embryos (Fig. 1C). A Vangl2 doublet that is present in wild type and *vangl2^ΔTMs/WT^* Western blots likely reflects the different phosphorylated states of Vangl2 [32]. Although the amino terminus is not encoded by exon 4, no smaller mutant isoforms of Vangl2 were detected in lysates from heterozygotes or knockouts. In addition mutant *vangl2* cDNAs were synthesized, cloned from *vangl2^ΔTMs/ΔTMs^* tissues and sequenced. None of these cDNAs encoded alternative mutant protein isoforms that could escape detection by the Vangl2 N-13 antibody (data not shown). Therefore *vangl2^ΔTMs^* is a complete null allele.

*Vangl2^ΔTMs^* mice were backcrossed to C57Bl6 for at least 5 generations before experimental intercrosses. On this genetic background the majority of *vangl2^ΔTMs/ΔTMs^* mutants exhibited craniorachischisis, a severe NTD (Fig. 1D–G) that is a characteristic phenotype of *Lp* mutants [22]. However, unlike *vangl2^Lp/Lp^*, 26% of *vangl2^ΔTMs/ΔTMs^* mice (6/23) present only with spina bifida, a milder NTD restricted to the posterior neural tube adjacent to the tail (Fig. 1F–G). Similarly, on the C57Bl6 background only 11% of *vangl2^ΔTMs/WT^* mice (11/99) had the tail loops or kinks that are characteristic of the *Lp* line. Tail phenotypes were never observed in *vangl2^ΔTMs/WT^* mice backcrossed for a minimum of 5 generations to the FVB (0/66 heterozygotes) or strain A/J (0/15 heterozygotes) inbred lines. Finally in some *vangl2^ΔTMs/ΔTMs^* KOs, incomplete or failed eyelid closure was observed that was similar in appearance to that described for other PCP mutants [3], [8]. However this was highly variable with some eyelids only partially closed and some animals showing unilateral penetrance. As a result this feature was not quantified. Together these observations suggest that aspects of the mutant phenotype are milder in *vangl2^ΔTMs/ΔTMs^* mice than in *vangl2^Lp/Lp^* mutants.

**Figure 2:**
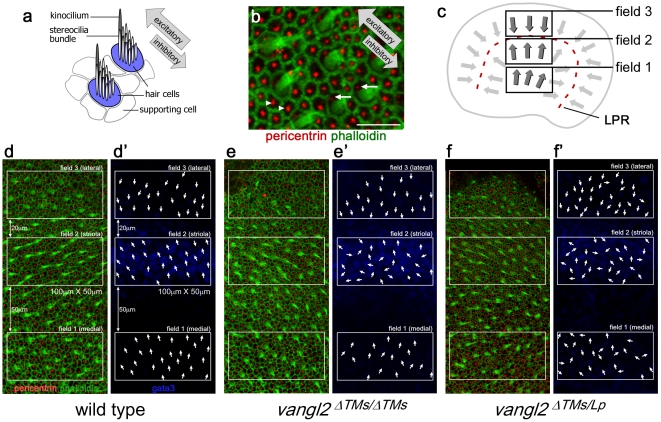
Vestibular hair cell planar polarity phenotypes are enhanced in *vangl2^ΔTMs/Lp^* utricle relative to *vangl2^ΔTMs/ΔTMs^*. (**A**) A polarized bundle of stereocilia is located adjacent to the kinocilium on the apical surface of vestibular hair cells. As indicated by annotated gray arrows, movements of the bundle towards the kinocilium produce excitatory electrophysiological responses while movements away are inhibitory. (**B**) Planar polarity is visualized by labeling the stereocilia with phalloidin (green) and the basal body beneath the kinocilium with anti-pericentrin antibodies (red). Pericentrin labeling also marks the position of a primary cilium on the intervening supporting cells which lack stereocilia. White arrows highlight single basal bodies in hair cells and arrowheads show single basal bodies in supporting cells. (**C**) Schematic illustration of the range of hair cell polarities (gray arrows), their organization about the line of polarity reversal (LPR, red dashed line), and the position of three analysis fields used throughout this study. (**D**) Wild type utricle hair cells labeled for planar polarity analysis with phalloidin (green) and pericentrin antibodies (red), and the position of the three analysis fields. (**D′**) All samples are also labeled for Gata3 (blue) to mark the striola region for analysis field placement (see Methods). For illustration purposes, individual hair cell orientations have been annotated based upon the labeling in (D) and have been superimposed on the Gata3 micrograph (D′). (**E,E′**) Utricles from *vangl2^ΔTMs/ΔTMs^* mice have misoriented hair cells that are restricted to analysis field2. (**F,F′**) Utricles from *vangl2^ΔTMs/Lp^* mice have misoriented hair cells throughout fields1&2. Scale bar for (B) is 10 µm. The dimensions of the analysis fields are all 100 µm×50 µm.

The *vangl2^ΔTMs/ΔTMs^* phenotype was assayed in greater detail in the inner ear because the sensory hair cells have distinct planar polarity that is readily visualized and quantified for direct comparison with the *Lp* mutant phenotype. The apical surface of a hair cell extends a bundle of stereocilia that is organized in a staircase pattern with the tallest stereocilia adjacent to single kinocilium. Planar polarity is apparent in the shared stereocilia∶kinocilium polarity of neighboring cells (Fig. 2A–B). Vestibular hair cells in the utricle are further divided between two groups separated by a line of polarity reversal (LPR, Fig. 2C), an organization that is analogous to the pattern of ommatidia in the *Drosophila* compound eye [33]. The LPR is located adjacent to a specialized region called the striola, and this region can be labeled with antibodies against the transcription factor Gata3 or the calcium binding protein Oncomodulin for use as an anatomical landmark (Fig. 2D–F, Fig. S1) [34], [35].

For polarity analyses the utricular maculae was divided into three analysis fields positioned about the immunolabeled striola. Field1 was located in the medial utricle, field2 encompassed the striola and was adjacent to the LPR, while field 3 was in the lateral utricle and contained hair cells of opposite orientation to fields 1&2 (Fig. 2C). Misoriented vestibular hair cells were identified in *vangl2^ΔTMs/ΔTMs^* utricles using phalloidin to mark the stereocilia and antibodies against pericentrin to label the basal body beneath the kinocilium (Fig. 2B,D–E). Affected hair cells were restricted to field2 while the organization of hair cells in fields 1 and 3 were similar to wild type controls. This contrasts with the number and distribution of misoriented vestibular hair cells in *vangl2^Lp/Lp^* mutants, where affected cells are reported throughout the utricular maculae [7].

In order to determine if this difference is due to a dominant phenotype resulting from the *Lp* mutation, the two mouse lines were intercrossed and the orientation of utricular hair cells was analyzed in *vangl2^ΔTMs/Lp^* embryos (Fig. 2F). If the *Lp* mutation is dominant, then the *vangl2^ΔTMs/Lp^* phenotype should be more severe than the *vangl2^ΔTMs/ΔTMs^* phenotype. Alternatively if *Lp* is recessive or hypomorphic then the *vangl2^ΔTMs/Lp^* phenotype should be the same or less severe than *vangl2^ΔTMs/ΔTMs^*. Prior to these intercross experiments, *Lp* mice were backcrossed to C57Bl6 for a minimum of four generations. Unfortunately after five backcross generations, male *vangl2^Lp/WT^* offspring were infertile and it was not possible to produce a pure congenic line.

**Figure 3:**
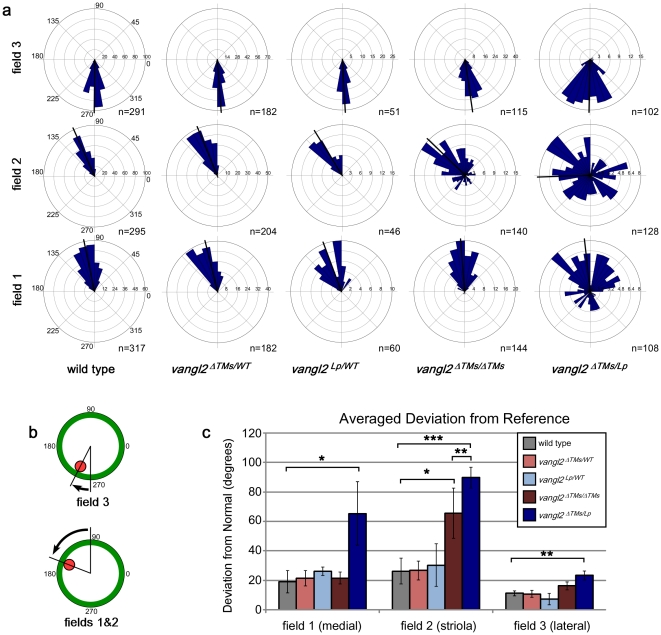
Quantification of vestibular hair cell orientation in control and *vangl2* mutant utricles. (**A**) Circular histograms demonstrating the orientations of all of the vestibular hair cells that were measured from fields1,2&3 for each of the genotypes listed. The number of cells in each bin is graphed along the x-axis and the cell total is listed. The average orientation of each group of cells is marked by a bold black line. (**B**) The averaged mean deviation of stereocilia bundle orientations from 0° was determined by measuring the absolute value of the angle formed by the bundle axis and a reference line drawn perpendicular to the striola. In this schematic red indicates the position of the kinocilium and green is the cell periphery. (**C**) The averaged deviation from 0° for hair cells located in fields1,2&3 for each experimental and control genotype. Error bars indicate standard deviation. Statistical significance was calculated by Student's t-test with unequal variance. (*P<0.05, **P<0.001, ***P<10^−5^).

In *vangl2^ΔTMs/Lp^* compound mutants, both the number of misoriented cells and the extent of their disorganization were increased in comparison to *vangl2^ΔTMs/ΔTMs^*. This included the appearance of misoriented cells in field1 and increased disorganization throughout field2. These trends were quantified by graphing the orientation (from 0–360°) of individual hair cells from all embryos using circular histograms (Fig. 3A), and by measuring the average absolute value of bundle orientations relative to a reference drawn perpendicular to the three analysis fields (Fig. 3B). Each approach demonstrated a significant difference in bundle orientation between *vangl2^ΔTMs/ΔTMs^* and controls in field1, and between *vangl2^ΔTMs/Lp^* and controls throughout the utricle (Fig. 3A,C). Moreover, in each of the three fields, a larger proportion of hair cells were misoriented in *vangl2^ΔTMs/Lp^* than in *vangl2^ΔTMs/ΔTMs^* mice, although orientation was least affected in field3 for all genotypes with only moderate changes in *vangl2^ΔTMs/Lp^*. Together these observations reveal a stronger mutant phenotype in *vangl2^ΔTMs/Lp^* mice and demonstrate potential dominant effects of the *Lp* mutation during hair cell development.

**Figure 4:**
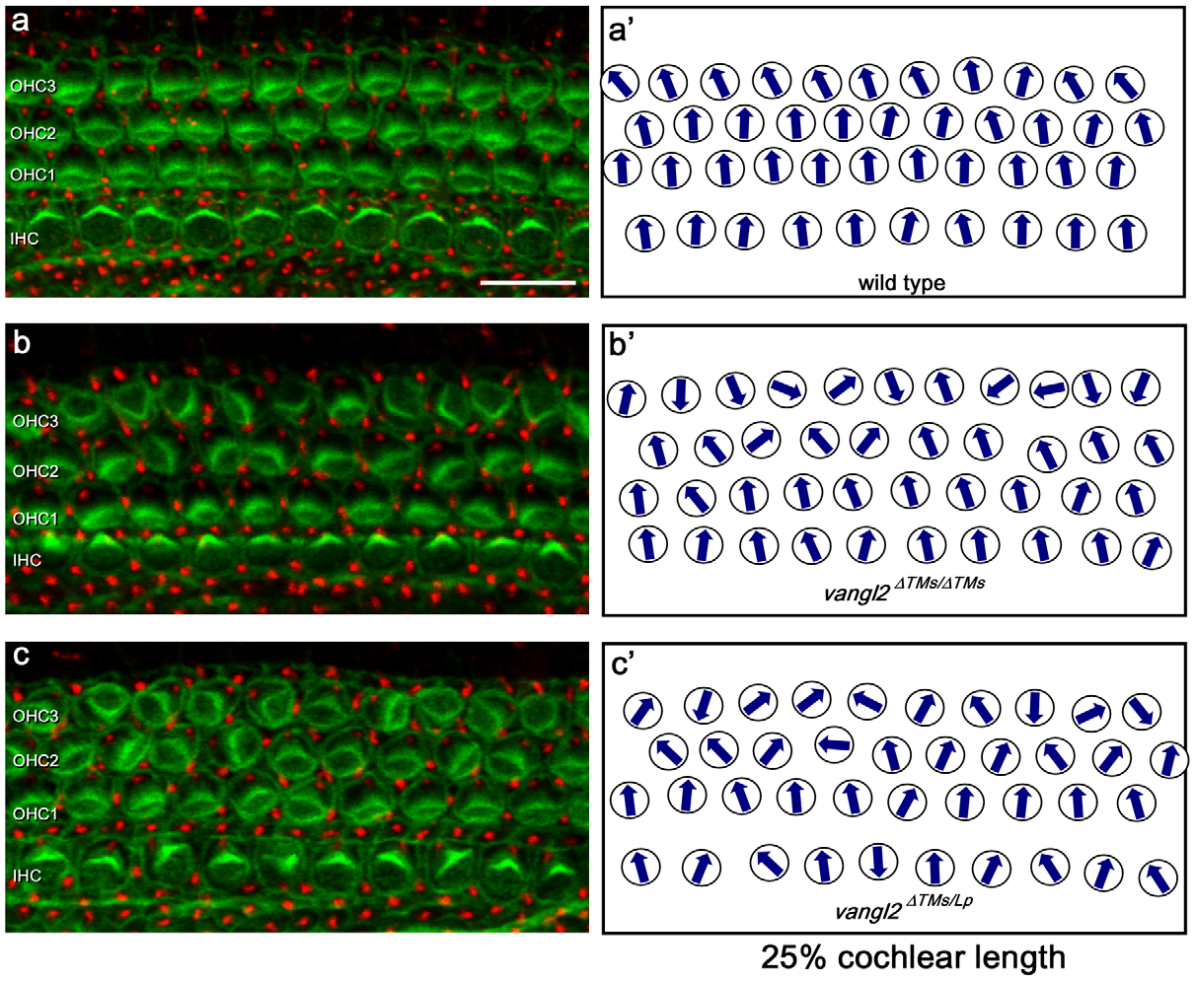
Auditory hair cell planar polarity phenotypes are enhanced in the *vangl2^ΔTMs/Lp^* cochlea relative to *vangl2^ΔTMs/ΔTMs^*. (**A**) Confocal image of auditory hair cells from an E18.5 wild type mouse in which hair cells are labeled for phalloidin (green) and pericentrin (red). (**A′**) Schematic illustrating the orientation of stereocilia bundles from each of the hair cells present in panel (A). (**B–C**) Confocal images and corresponding orientation schematics from *vangl2^ΔTMs/ΔTMs^* (B,B′) and *vangl2^ΔTMs/Lp^* cochleae (C,C′). All images were collected from a basal position corresponding to 25% of the length of the cochlea as measured from the base to the apex. IHC (inner hair cell), OHC1 (outer hair cell 1), OHC2 (outer hair cell 2), OHC3 (outer hair cell 3). Scale bar is 10 µm.

**Figure 5:**
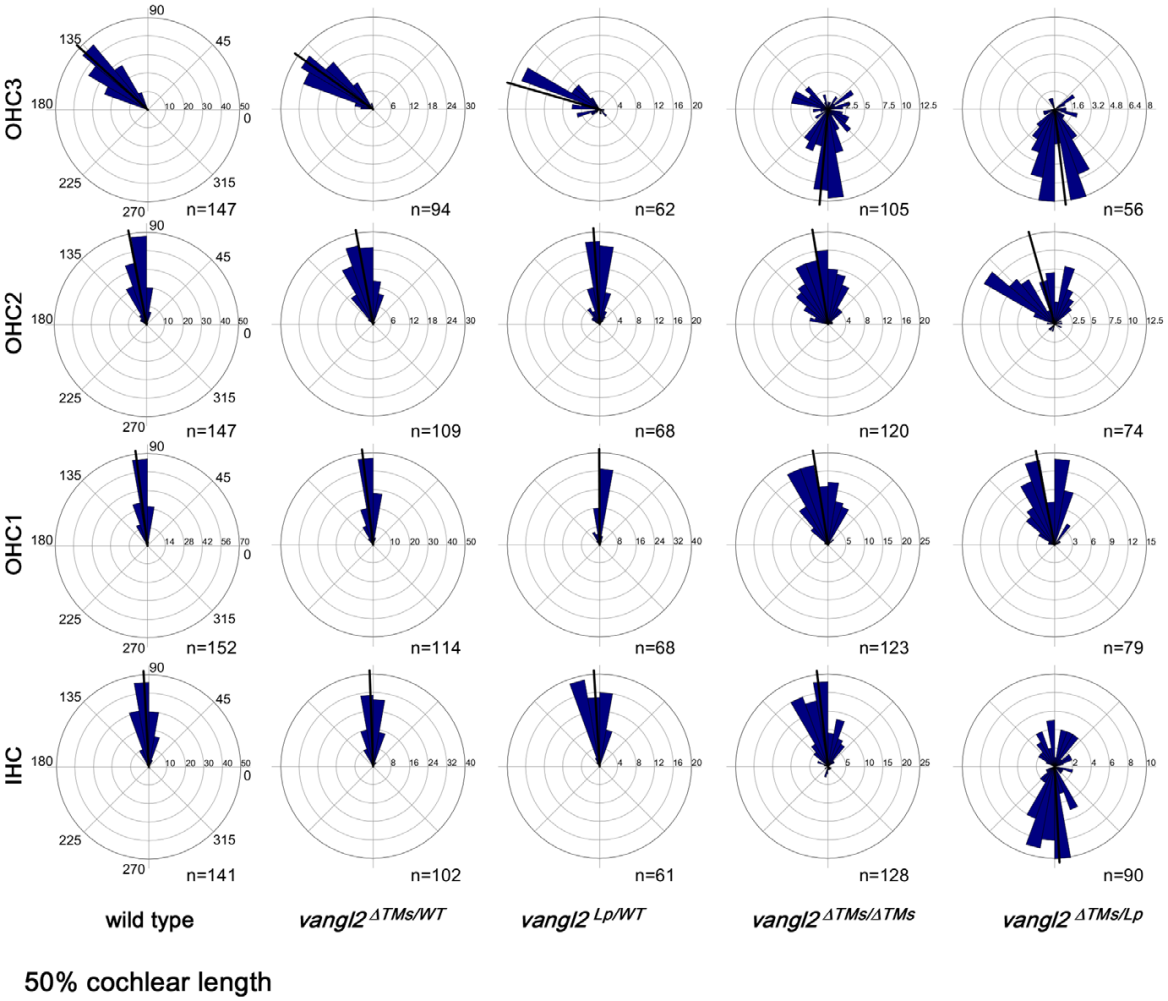
Circular histograms quantifying the orientation of all auditory hair cells. The orientation of individual auditory hair cells was measured at a position corresponding to 50% of the cochlear length based upon phalloidin and pericentrin immunolabeling. Hair cells from each row (IHC, OHC1, OHC2, OHC3) were analyzed separately, and each column of histograms corresponds to the separate genotypes listed beneath. The number of cells in each bin is graphed along the x-axis and the total number of cells for each histogram is listed. For these histograms 90° is pointed away from the spiral ganglion and 180° is pointed towards the apex of the cochlea. The average orientation of each group of cells is marked by a bold black line.

**Figure 6:**
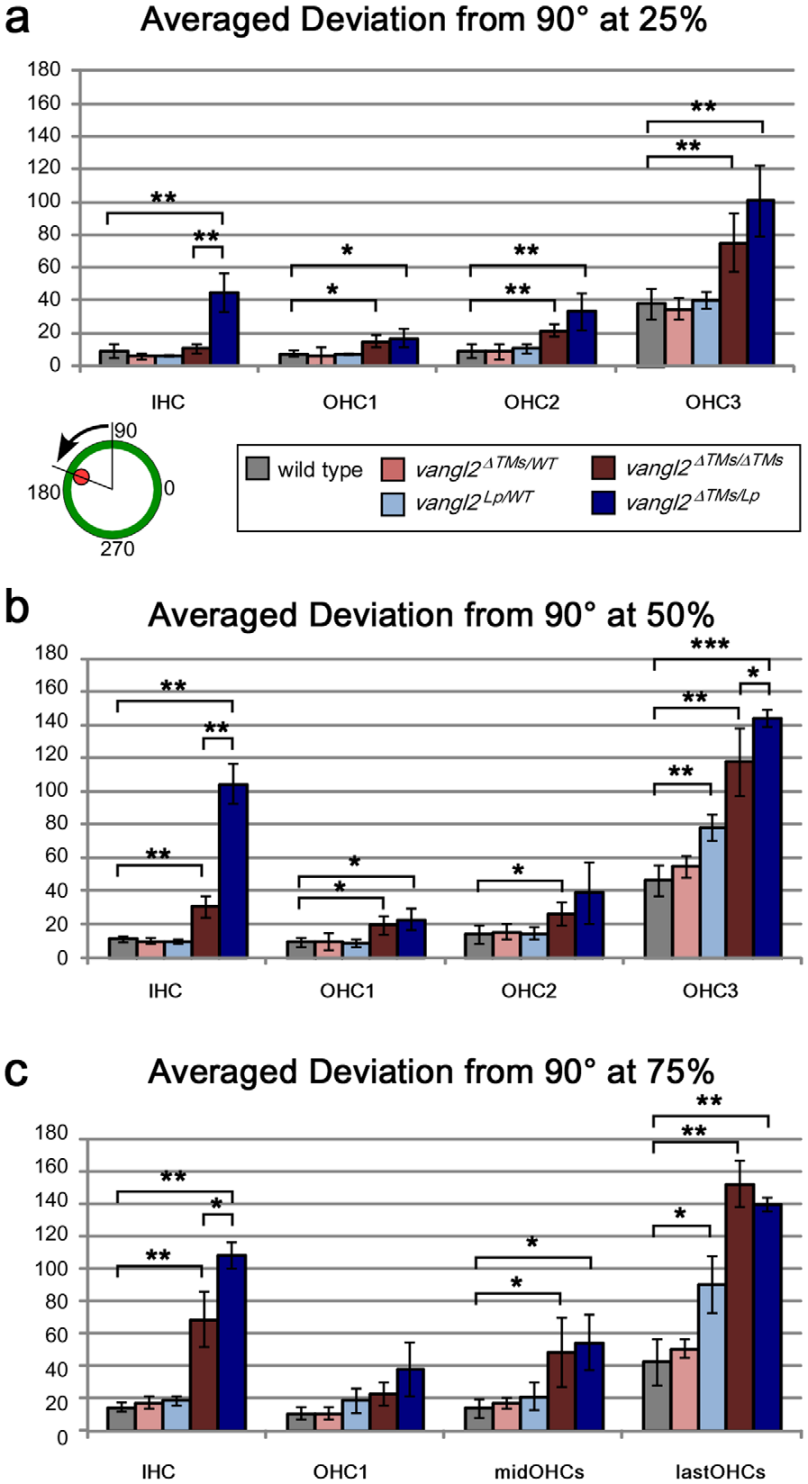
Quantification of averaged stereocilia bundle orientation for auditory hair cells. (**A–C**) The averaged mean deviation of stereocilia bundle orientation was determined by measuring the absolute value of the angle formed by the bundle axis and a reference line drawn perpendicular to the 4 rows of hair cells. (**A**) In this schematic red indicates the position of the kinocilium and green is the cell periphery. The averaged deviation was graphed separately for positions located at (**A**) 25%, (**B**) 50%, and (**C**) 75% the length of the cochlea as measured from the base to the apex. For some mutant samples, additional rows of outer hair cells were present at the 75% position. To facilitate comparisons between genotypes, all hair cells located between OHC1 and the last row of hair cells (lastOHCs) are combined and graphed as MidOHCs (middle rows of OHCs). Error bars indicated standard deviation. Statistical significance was calculated by Student's t-test with unequal variance. (*P<0.05, **P<0.001, ***P<10^−5^).

The planar polarity of auditory hair cells was also disrupted in the cochlea of *vangl2^ΔTMs/ΔTMs^* KOs (Fig. 4–6). In the cochlea, a progression of hair cell differentiation and polarization occurs along the length from the base to the apex. As a result, more mature stereocilia bundles are present on hair cells positioned in regions closer to the base (analyzed at 25% of the cochlear length) than in apical regions (analyzed at 75% of the cochlear length). In addition, morphogenesis of outer hair cells (OHCs) initiates after inner hair cells (IHCs). Consequently at E18.5, OHC3 stereocilia bundles are still transiently oriented towards the apical tip of the cochlea (see wild type OHC3, Fig. 5) [36]. OHC3 is also the most susceptible to mutations in PCP genes. Consistent with this, OHC3 is most affected in the *vangl2^ΔTMs/ΔTMs^* mice, with misoriented cells in this row along the length of the cochlea (Fig. 6). IHC orientation is also altered in *vangl2^ΔTMs/ΔTMs^* mice but primarily in the less mature apical positions (Fig. 6B–C).

The quantification of auditory hair cell polarity in *vangl2^ΔTMs/Lp^* mice provides additional evidence that the *Lp* mutation is dominant. Similar to the utricle, a larger proportion of auditory hair cells are affected than in *vangl2^ΔTMs/ΔTMs^* or controls, and within a given row of cells there is an increase in the extent of disorganization. This can be seen for IHCs labeled with phalloidin and pericentrin in the base of the cochlea (Fig. 4). This is further evident when the orientations of all cells are graphed in circular histograms (Fig. 5), and when the average absolute value of bundle orientations is measured relative to neural to abneural axis at three separate points along the length of the cochlea (Fig. 6). Overall the greatest difference between *vangl2^ΔTMs/Lp^* and *vangl2^ΔTMs/ΔTMs^* phenotypes occurs for IHCs, and this difference can be measured at all points along the length of the cochlea. It should also be noted that a statistically significant difference in averaged bundle orientation can be detected between *vangl2^Lp/WT^* heterozygotes and *vangl2^ΔTMs/WT^* or wild type control littermates (Fig. 6B–C). Although this difference is only detected for OHC3 hair cells in the apical turns of the cochlea, it is consistent with a partially penetrant and dominant *Lp* phenotype. In comparison no differences were seen between *vangl2^ΔTMs/WT^* and wild type hair cells.

In contrast to vestibular hair cells in the utricle, the orientation of auditory hair cells in *vangl2^ΔTMs/Lp^* mutants does not appear random. Instead a large proportion of IHCs and OHC3s appear to be reversed by 180 degrees (Fig. 5). This is similar to the reversed orientation of IHCs observed in *fz3/6* DKOs [8] and may reflect the activity of residual polarity mechanisms that function in parallel to core PCP signals. In addition, and as expected based upon previous *Lp* analyses [11], a portion of the *vangl2^ΔTMs/ΔTMs^* and *vangl2^ΔTMs/Lp^* cochleae analyzed had extra rows of outer hair cells at the 75% position. In these samples the orientation of stereocilia bundles for OHC3 was not significantly altered and was more similar to OHC2 than the outermost row of ectopic cells. Therefore for quantification purposes, all hair cells located between OHC1 and the last row of outer hair cells (lastOHCs) were grouped and analyzed together as the middle rows (midOHCs, Fig. 6). It was unclear whether the appearance of these rows was due to defects in convergent extension movements or was secondary to craniorachischisis in these mutants.

Together these experiments suggest that the presence of the Vangl2^S464N^ mutant protein has a greater effect on cellular polarization than the loss of wild type Vangl2. Recently it was shown that the *Lp* mutation disrupts Vangl2 association with Sec24b, a trafficking protein required for export from the endoplasmic reticulum [27]. This mechanism may also be the basis of craniorachischisis in *Lp^m1Jus^*, a second *vangl2* mutant line with an aspartic to glutamic acid substitution at amino acid position 255 (Vangl2^D255E^) [29]. As a result, in each of these mutants, Vangl2 protein is retained within the endoplasmic reticulum and fails to be delivered to the plasma membrane. Two separate mutations in Sec24b also disrupt Vangl2 trafficking resulting in similar craniorachischisis phenotypes [27], [28].

One explanation for the stronger phenotype that occurs in *vangl2^ΔTMs/Lp^* mutants than *vangl2^ΔTMs/ΔTMs^* KOs is that altered Vangl2^S464N^ trafficking disrupts the distribution of other polarity molecules. A likely candidate is Vangl1 because Vangl1 expression overlaps with Vangl2, and enhanced planar polarity defects are seen in mice lacking both *vangl1* and *vangl2* [12], [37]. Remarkably Van Gogh proteins oligomerize into larger protein complexes in *Drosophila* [38] raising the possibility that if similar oligomeric complexes are formed in vertebrates, then Vangl2^S464N^ or Vangl2^D255E^ may disrupt Vangl1 or Vangl2 delivery to the plasma membrane. This hypothesis was tested using a heterologous system in which hemagluttinin (HA) tagged Vangl2 constructs were co-expressed with EGFP-tagged Vangl1 or Vangl2. Specifically these experiments utilized 3XHA-tagged Vangl2, 3XHA-Vangl2^S464N^ and 3XHA-Vangl2^D255E^ constructs characterized by Merte *et al*. [27].

**Figure 7:**
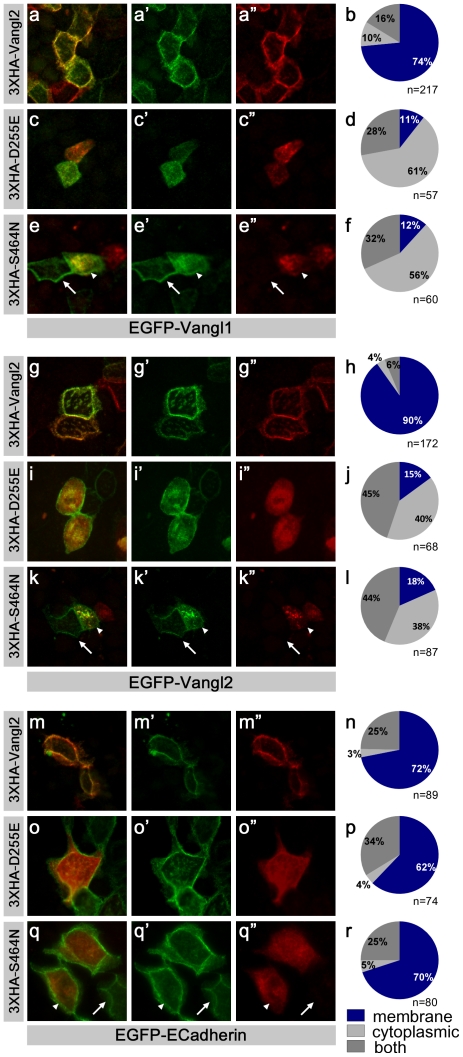
Mutant Vangl2 protein alters the distribution wild type Vangl1 and Vangl2. (**A–F**) The effect of mutant Vangl2 proteins on the distribution of Vangl1 was determined by co-expressing EGFP-Vangl1 in MDCK cells together with 3XHA Vangl2 (A–B), 3XHA Vangl2^D255E^ (C–D) or 3XHA Vangl2^S464N^ (E–F). Recombinant protein distribution was visualized after 72 hours in culture using antibodies against GFP (green) or HA (red). (**G–L**) Similarly the effect of mutant Vangl2 proteins on the distribution of wild type Vangl2 was determined by co-expression of EGFP-Vangl2 with 3XHA Vangl2 (G–H), 3XHA Vangl2^D255E^, (I–J) or 3XHA Vangl2^S464N^ (K–L). (**M–R**) Mutant Vangl2 proteins do not disrupt the membrane localization of E-Cadherin-GFP. (E,K,Q) Representative cells only expressing EGFP-Vangl1, EGFP-Vangl2 or E-Cadherin-GFP are marked by arrows and representative cells co-expressing 3XHA Vangl2 ^S464N^ and EGFP-constructs are marked by arrowheads. (**B,D,F,H,J,L,N,P,R**) The subcellular distribution of EGFP in co-transfected cells was scored as membrane associated (blue), cytoplasmic (light gray), or both membranous and cytoplasmic (gray) and graphed. Quantification was completed for cells obtained from one round of electroporations and the total numbers of co-transfected cells analyzed for each condition are indicated beneath the pie chart. These findings were consistent with additional electroporations experiments in which cells were imaged but not quantified.

Consistent with previous reports, following electroporation into MDCK cells, the control constructs 3XHA-Vangl2, EGFP-Vangl2 and EGFP-Vangl1 were delivered to the plasma membrane and were enriched at cell boundaries (Fig. 7A–B,G–H, Fig. S2). This distribution was enhanced 96 hours post-electroporation, presumably due to the maturation of intercellular junctions between MDCK cells (data not shown). However there was never an asymmetric redistribution of Vangl1 or Vangl2 fusion proteins in the cultured cells. In contrast, 3XHA-Vangl2^S464N^ or 3XHA-Vangl2^D255E^ constructs were found predominantly in cytoplasmic compartments (Fig. 7C,E,I,K,O,Q) similar to the distribution of the mutant proteins in COS cells [27]. Vangl2 mutant proteins were also less stable than wild type with a half life of approximately 48 hours. Therefore protein distribution studies were conducted 72 hours post-electroporation when the majority of wild-type proteins were present at cell boundaries (Fig. 7B,H,N). At this time co-expression of 3XHA-Vangl2^S464N^ or 3XHA-Vangl2^D255E^ significantly changed the subcellular distribution of EGFP-Vangl1 (Fig. 7A–F) and EGFP-Vangl2 (Fig. 7G–L). In the presence of mutant Vangl2, detectable EGFP-Vangl1 or EGFP-Vangl2 protein was reduced at the membrane and increased in intracellular compartments. Furthermore the distribution of EGFP-Vangl1 (arrowheads, Fig. 7E) or EGFP-Vangl2 (arrowheads, Fig. 7K) appeared similar to mutant Vangl2 within cells expressing both constructs. This effect specifically occurred between Vangl proteins as 3XHA-Vangl2^S464N^ and 3XHA-Vangl2^D255E^ did not alter the distribution of E-Cadherin-GFP, another membrane spanning protein enriched at MDCK cell boundaries (Fig. 7M–R). MDCK cell electroporation with the tagged constructs also did not alter the distribution of endogenous E-cadherin (Fig. S2).

**Figure 8:**
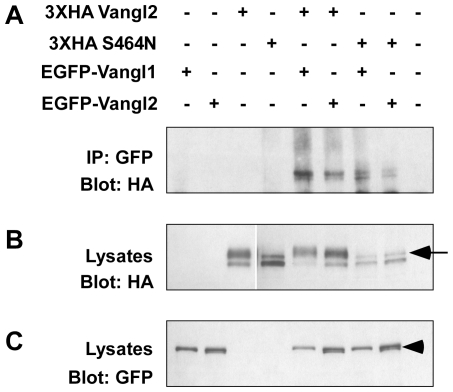
Co-Immunoprecipitation assays show oligomeric complex formation consisting of EGFP-Vangl1 and 3XHA-Vangl2, or EGFP-Vangl2 and 3XHA-Vangl2. (**A**) Immunoprecipitation (IP) of EGFP-Vangl1 or EGFP-Vangl2 using an antibody against GFP pulls down 3XHA-tagged Vangl2 when co-expressed in MDCK cells demonstrating the formation of an oligomeric complex. Similarly, EGFP-Vangl1 or EGFP-Vangl2 IPs also pull down mutant 3XHA-Vangl2^S464N^ protein showing that the *Looptail* mutation does not disrupt oligomerization. (**B,C**) Western blots of MDCK cell lysates alone were used to confirm expression of HA-tagged (B) and EGFP-tagged constructs (C), and equilibrate the amount of cell lysates used for immunoprecipitation. Phosphorylated 3XHA-Vangl2 runs in a slower migrating band (arrow, B) and phosphorylated EGFP-Vangl2 generates a doublet (arrowhead, C) in denaturing SDS-PAGE gels of immunoprecipitates or cell lysates. Phosphorylation of 3XHA-Vangl2^S464N^ mutant protein is decreased under all conditions.

The potential for physical interactions between Vangl1 and Vangl2, and between different Vangl2 molecules were further assayed by co-immunoprecipitation of 3XHA and EGFP-tagged proteins from MDCK cells. Using this approach, immunoprecipitation of EGFP-Vangl1 or EGFP-Vangl2 with GFP antibodies pulled down 3XHA-Vangl2 when the constructs were co-expressed (Fig. 8A). Similarly EGFP-Vangl1 and EGFP Vangl2 bound and co-precipitated 3XHA-Vangl2^S464N^. Doublets in HA (Fig. 8B) and EGFP blots (Fig. 8C) demonstrate that the phosphorylation of tagged-Vangl2 proteins is similar to wild type (Fig. 1C and [32]). Altogether these *in vitro* studies utilizing MDCK cells argue that the molecular basis of dominant *Lp* mutations is the disrupted trafficking of multimeric Vangl1/2 complexes resulting in an overall reduction in planar polarity.

**Figure 9:**
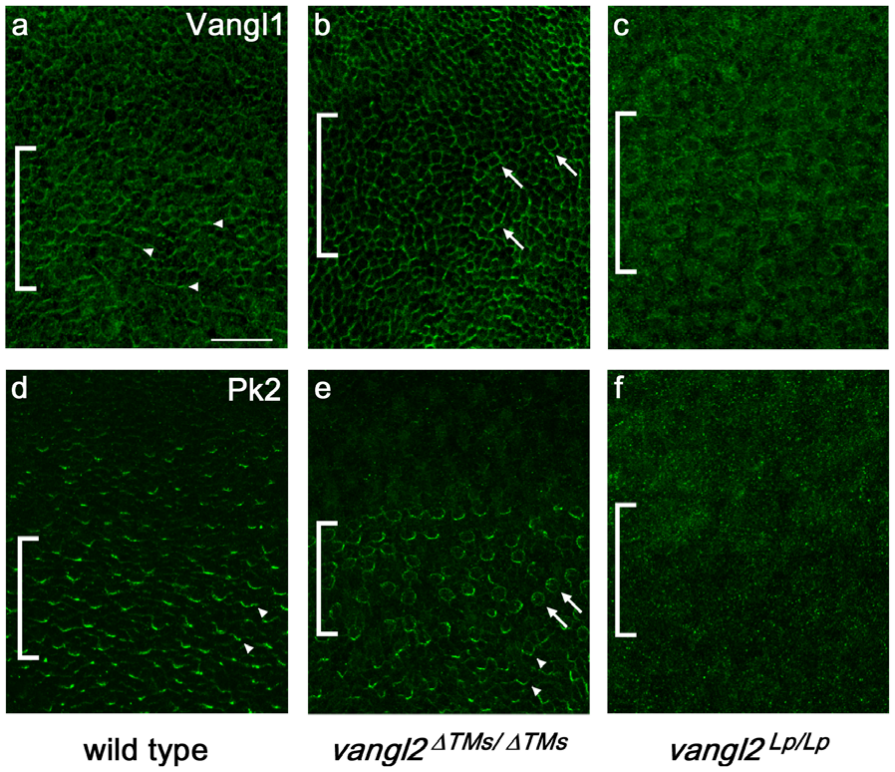
Subcellular distributions of PCP proteins are differentially affected in *vangl2^ΔTMs/ΔTMs^* versus *vangl2^Lp/Lp^* mutant utricles. (**A**) Vangl1 immunolabeling is enriched at cell boundaries in the wild type utricular maculae, within 3–6 µm of the cell surface as shown by confocal microscopy. Asymmetric protein localization is evident at many cell boundaries (arrowheads). (**B**) Apical localization of Vangl1 is maintained in the *vangl2^ΔTMs/ΔTMs^* utricle although asymmetric localization is lost, and individual hair cells are frequently surrounded by Vangl1 protein (arrows). (**C**) Vangl1 protein is significantly reduced from apical cell boundaries in *vangl2^Lp/Lp^* utricles. (**D**) Pk2 is enriched at hair cell:support cell boundaries throughout the wild type utricle (examples marked by arrowheads). (**E**) In *vangl2^ΔTMs/ΔTMs^* tissue, the distribution of Pk2 changes in a region specific manner. In the bracketed striola region, Pk2 protein frequently surrounds individual hair cells (arrows). In lateral regions apical protein localization is lost, while Pk2 is maintained in a normal pattern in the medial utricle (arrowheads). (**F**) Similar to changes in Vangl1 distribution, the presence of Pk2 at apical cell boundaries is lost throughout the *vangl2^Lp/Lp^* utricle. Brackets indicate the approximate positions of the striola region based upon Oncomodulin labeling in a separate channel. Scale bar is 25 µm.

To determine if the *Lp* mutation disrupted Vangl1 distribution *in vivo*, wholemount immunolabeling was used to visualize Vangl1 protein distribution in *vangl2^ΔTMs/ΔTMs^* and *vangl2^Lp/Lp^* utricles. Although Vangl1 and Vangl2 are 71% identical (NCBI pair-wise BLAST), the Vangl1 antibody appears specific for Vangl1 because there are no differences in Western blot analyses between protein lysates collected from WT and *vangl2^ΔTMs/ΔTMs^* embryos (Fig. S3A). Despite this, the antibody cannot distinguish EGFP-Vangl1 and EGFP-Vangl2 by Western blot when the EGFP-tagged proteins are over-expressed in MDCK cells. Still there is a 10-fold greater affinity of the antibody for EGFP-Vangl1 than EGFP-Vangl2 (Fig. S3B). Vangl1 immunolabeling of the mouse utricle is enriched at the apical surface of vestibular hair cells, and labeling is frequently asymmetric at boundaries (Fig. 9A, arrowheads). Vangl1 immunolabeling is maintained at the apical cell surface in *vangl2^ΔTMs/ΔTMs^* utricles; however Vangl1 distribution frequently appears more uniform and often surrounds mutant hair cells (Fig. 9B, arrows). For these experiments the position of the striola region was determined by Oncomodulin immunolabeling in a second channel (data not shown) and is indicated by brackets (Fig. 9 A–F). In dramatic contrast, the apical localization of Vangl1 protein fails to occur throughout all regions of the *vangl2^Lp/Lp^* mutant utricle (Fig. 9C). Instead fluorescent puncta are visible which may reflect the redistribution of Vangl1 within intracellular organelles. These *in vivo* results are consistent with those *in vitro*, and support the hypothesis that disrupted trafficking of *Lp* mutant Vangl2^S464N^ protein inhibits delivery of Vangl1 to the cell surface.

To establish whether altered Vangl2 trafficking, or the resulting changes in planar polarity, affected the behavior of other PCP proteins, the distribution of Prickle 2 (Pk2, Entrez Gene ID: 243548) was assayed in *vangl2^ΔTMs/ΔTMs^* and *vangl2^Lp/Lp^* utricles. In *Drosophila*, Prickle binds to Van Gogh and the two proteins are asymmetrically localized to the proximal side of wing epidermal cells [39] where Prickle promotes the intracellular amplification of cellular polarity [40]. In the mouse, Pk2 is also asymmetrically localized in hair cells and support cells throughout the vestibular epithelia (Fig. 9D, arrowheads, and [5]). In vertebrates, Pk2 localization is likely to require intact PCP signaling rather than direct interactions with Vangl2 because EGFP-Pk2 appears cytoplasmic in MDCK cells and apical or asymmetric localization is not enriched with co-expression of 3× HA-Vangl2 (data not shown). Nonetheless, Pk2 localization *in vivo* is altered in both *vangl2^ΔTMs/ΔTMs^* and *vangl2^Lp/Lp^* tissues; although the nature of the changes differs between the two genotypes. In *vangl2^ΔTMs/ΔTMs^* KOs, the polarized distribution of Pk2 is lost in the striola, where Pk2 is maintained at the apical cell surface but frequently encircles individual hair cells (Fig. 9E, arrows). Cells in this region frequently have misoriented bundles (see Fig. 2E). Asymmetric Pk2 localization is maintained in the medial utricle (Fig. 9E, arrowheads) yet curiously, Pk2 is absent from all cells in the lateral region. In *vangl2^Lp/Lp^* mutant utricle by comparison, the apical localization of Pk2 is completely lost (Fig. 9F) similar to previous reports [5]. This effect on Pk2 in *vangl2^Lp/Lp^* mutants is likely due to the overall disruption of cellular polarity that is secondary to changes in the distribution of Vangl1 and Vangl2. Consistent with the previous *in vitro* findings, these *in vivo* results further demonstrate that the *Lp* mutation exerts dominant phenotypic effects by disrupting the molecular polarization of developing cells.

## Discussion

By generating a novel *vangl2* knockout line and intercrossing those mice with *Lp* mutants, we have demonstrated the dominant nature of the *Lp* mutant phenotype during inner ear development. This was completed through a detailed quantification and comparison of the planar polarity phenotypes of hair cells in *vangl2^ΔTMs/Lp^* and *vangl2^ΔTMs/ΔTMs^* mice. In addition we propose a mechanism for the dominant effect of the *Lp* mutation in which disrupted trafficking of Vangl2^S464N^ alters the distribution of Vangl1, Vangl2 and Pk2. Together these findings will guide interpretation of studies employing the *Looptail* line, particularly when *Looptail* mice are used for molecular dissections of the PCP signaling pathway.

At a molecular level, the decreased protein stability and cell-surface presentation of Vangl2^S464N^ is due to disrupted trafficking of the mutant protein and its retention in the ER [27]. We have demonstrated that co-expression of Vangl2^S464N^ with Vangl1 in a heterologous system also prevents Vangl1 delivery to the cell surface. Since *vangl1* and *vangl2* regulate planar polarity in many tissues including the inner ear [12], [13], it stands to reason that the combined loss of Vangl1 and Vangl2 would increase hair cell orientation defects. Consistent with this hypothesis, planar polarity defects are less in those *vangl2^ΔTMs/ΔTMs^* vestibular hair cells that maintain apical localization of Vangl1 than in *vangl2^Lp/Lp^* hair cells where apical Vangl1 is lost. We also demonstrate that Vangl2 forms oligomers with Vangl1 and Vangl2 in heterologous cells. This is similar to findings from *Drosophila* where Van Gogh oligomerizes *in vivo* [38]. Many integral membrane proteins are folded and assembled into oligomeric structures in the ER and inappropriate assemble or transport out of the ER results in protein degradation by “quality control” systems [41], [42]. Therefore our results, together with these previous findings, suggest that the Vangl2^S464N^ protein encoded by the *Lp* mutation exerts dominant effect on planar polarity by inhibiting the membrane trafficking and localization of Vangl1 and Vangl2, likely leading to their degradation.

This mechanism is sufficient to explain the milder neural tube phenotype that occurs in *vangl2^Lp/WT^* heterozygotes if the presence of Vangl2^S464N^ reduces but does not eliminate all Vangl1/2 protein from the cell surface. By comparison the uterine epithelium of the female reproductive track (FRT) is more sensitive to the *Lp* mutation than the neural tube. In a recent study where embryonic FRT tissues were cultured *in vitro*, similar developmental deficits were observed explants from *vangl2^Lp/WT^* and *vangl2^Lp/Lp^* embryos, further demonstrating the dominant effect of *Lp* [23]. Consistent with this study, we also found a mild planar polarity phenotype in the cochlea of *vangl2^Lp/WT^* heterozygotes that was restricted to OHC3 and was only present in the less developed apical turns (Fig. 6B–C). In contrast OHC3 is not affected in *vangl2^ΔTMs/WT^* cochlea. Although the molecular basis of Lp sensitivity was not established in the FRT, tissue specific responses to the *Lp* mutation in heterozygotes are likely determined by the complement of PCP proteins expressed in the tissue and their capacity to compensate for the presence of Vangl2^S464N^.

The severity of the *Lp* mutant phenotype is also influenced by genetic modifiers and can vary between different labs [3], presumably due to differences in husbandry practices and strain background. We have attempted to control this variable using two parallel approaches. First, the *vangl2^ΔTMs^* and *Lp* lines were maintained through successive heterozygote backcrosses with wild type C57Bl6 mice. This approach is limited however, because *vangl2^Lp/WT^* males are infertile after five backcross generations. In contrast, *vangl2^ΔTMs/WT^* mice can be backcrossed with C57Bl6 for at least nine generations without significant decreases in breeding potential. Second, by analyzing *vangl2^ΔTMs/Lp^* compound mutants rather than *vangl2^Lp/Lp^*, we sought to make heterozygous any genetic modifiers that may be unique to our colony of *Looptail* mice. Therefore the phenotypic differences between *vangl2^ΔTMs/Lp^* and *vangl2^ΔTMs/ΔTMs^* mutants are unlikely to be due to genetic modifiers. Overall these phenotypic comparisons combined with *in vitro* and *in vivo* protein localization studies demonstrate the dominant nature of the *Lp* mutation and provide a molecular basis for this effect.

The impact of the dominant nature of the *Lp* mutation on genetic interactions between this allele and other mutations should not be overlooked. However our results do not imply that conclusions drawn from previous intercrosses with *Lp* are false. In contrast, these findings demonstrate that crosses with *Lp* may represent a more sensitive assay of genetic interactions with the PCP signaling pathway than analogous crosses that could be generated using *vangl2^ΔTMs^*. Still it is important to distinguish between mutations that are merely permissive and only increase the penetrance of the *vangl2^Lp/WT^* phenotype versus mutations that may have an additive effect when combined with *vangl2^ΔTMs/ΔTMs^*, because the former genes may only be indirectly involved in planar polarity. Two additional *vangl2* mutations should also prove useful for genetic analyses. One is the *Lp-m1Jus* mutation that has similar molecular and phenotypic characteristics as the *Lp* mutation [27], [29]. The second is *Lp-m2Jus* which has a recessive phenotype with partially penetrant and hypomorphic characteristics in homozygotes such as looped tails and spina bifida [43]. Moreover the orientation of auditory hair cells is undisturbed in *Lp-m2Jus* mutants. Together the growing collection of *vangl2* mutant mouse lines could be employed as an allelic series for distinguishing subtler aspects of planar polarity during tissue development.

## Materials and Methods

### Ethics Statement

Mice were maintained at Harvard Medical School or The Johns Hopkins University School of Medicine under the corresponding IACUC-approved guidelines. The Johns Hopkins University School of Medicine Institutional Animal Care and Use Committee specifically approved this study under animal use protocols MO08M408 and MO11M394.

### *Vangl2* gene targeting

A gene targeting vector was assembled from mouse genomic DNA purified from the TC1 mouse ES cell line (P. Leder, Harvard Medical School). The vector contained a 4.2 kb 5′ arm that was modified by inserting LoxP and Hind3 sequences into a unique Xmn1 restriction site located in the intron upstream of *vangl2* exon 4. A second LoxP sequence and NeoR cassette flanked by FRT sequences was introduced into a unique BstB1 RE site located in the intron downstream of exon 4. The remainder of the vector consisted of a 5.1 kb 3′ homologous arm and DTA negative selection cassette. Mouse genomic DNA sequences used as homologous arms were amplified by PCR using the Roche Expand Long Template PCR System (Roche) and coding sequence exons located within the homologous arms were sequenced to check for PCR induced mutations. Following electroporation into the TC1 ES cell line and positive selection with G418, homologous recombination was validated in surviving clones by PCR and Southern blot (Fig. 1). A single ES cell line was injected into C57Bl6 blastocysts by the Brigham and Women's Hospital Transgenic Core Facility (Boston, MA). Following germline transmission, founders were crossed with transgenic mice ubiquitously expressing

FlpE recombinase (*ACTB-FlpE*) to remove the NeoR gene. The resulting allele (*vangl2^LoxP^ *) was not viable when homozygosed because the positions of the remaining LoxP sites disrupted *vangl2* expression. As a result, *vangl2^LoxP^* was crossed to transgenic mice ubiquitously expressing Cre recombinase (*ACTB-Cre*) to permanently delete exon 4 in the germ line and produce the *vangl2^ΔTMs^* knockout line.

### Mouse husbandry and genotyping

For general colony maintenance, *vangl2^ΔTMs/WT^* mice were crossed to mice from the C57Bl6 inbred line. For backcrossing, *vangl2^ΔTMs/WT^* female mice were bred to male mice from the C57Bl6, FVB or A/J inbred lines for a minimum of 5 generations prior to phenotypic analysis of heterozygotes or experimental intercross. The *Lp* mouse line was maintained by breeding with B6129S3F1/J hybrid mice and heterozygotes were identified based upon the presence of looped or kinked tails. Prior to experimental intercross with *vangl2^ΔTMs^*, the *Lp* mice were backcrossed to C57Bl6 for 4 generations. Additional backcrosses were not possible as BX+5 male mice were infertile and female mice had a high frequency of imperforate vagina that is common to this line. Mice from strains A/J, B6129S3F1/J, *ACTB-Cre* and the *Lp* mutant line were purchased from The Jackson Laboratory (Bar Harbor, ME). C57Bl6 and FVB mice were purchased from Charles River Laboratories (Wilmington, MA) and the *ACTB-FLPe* mice were obtained from S. Dymecki (Harvard Medical School).

*Vangl2^ΔTMs^* mice were genotyped using a common 5′ primer (5′ATCACCTCACTTGGCTGGAATAGATG 3′) paired with 3′ primers that were either KO-specific (5′GAAGTTATAAGCTTTGTTCCAG 3′) or WT-specific (5′GGCGAATGGGAGAAAGGCAGAC 3′). Following *vangl2^ΔTMs^* and *Lp* experimental intercross, the *Lp* mutation was genotyped by PCR amplification with primers flanking the mutation followed by DNA purification and sequencing of the amplified product. *Lp* genotyping primers are (5′ATATTTGGCTGCTGGACCCACCATCC3′) and (5′TGCAGCCGCATGACGAACTTATGTGA3′).

### Antibodies and Immunolabeling

Immunofluorescent labeling of auditory and vestibular hair cells was completed using E18.5 inner ears fixed for 2 hours in a solution of 4% paraformaldehyde prepared in Sorenson's phosphate buffer (pH7.4). Utricles and cochleae were subsequently removed, dissected to expose the surface of the sensory epithelia, permeabilized and blocked using blocking solution (5% donkey serum, 1% BSA, PBS) supplemented with Triton X-100 to 0.5%. Primary antibodies and phalloidin Alexa488 (Invitrogen A12379) were diluted in blocking solution supplemented with Tween-20 to 0.1%, and incubated with the tissue overnight at 4°C. Tissue was washed thoroughly with PBS-T (PBS, 0.05% Tween-20) followed by incubation with species-specific, Alexa Fluor (Invitrogen) or DyLight (Jackson ImmunoResearch) conjugated secondary antibodies. Tissue was subsequently washed with PBS-T, mounted using Fluoro-Gel (Electron Microscopy Sciences), and imaged using a Zeiss LSM510 confocal microscope. Image frames were limited to 1.5 microns in the Y-dimension and a stack of images 3–6 microns containing the stereocilia and apical surface of the hair cells was collected. A single image was generated by Z-projection using maximum or averaged pixel intensities. For preparations in which the striola region was marked using Gata3 antibodies, a second stack positioned deeper in the tissue was necessary to image Gata3 positive nuclei. The following commercial antibodies were used in this study: Actin (Millipore mAB1501), GFP (Invitrogen A11122), Gata3 (R&D AF2605), HA (Covance MMS-101P),Acetylated Tubulin (Sigma T7451), Vangl1 (Sigma A36455), Vangl2 (Santa Cruz SC46561), Oncomodulin (Santa Cruz SC7446), Pericentrin (Covance PRB432C). The Pk2 antibody was a gift from M. Scott and J. Axelrod (Stanford Univ.) and has been previously characterized [5].

### Quantification of hair cell planar polarity

For auditory hair cell analyses the organ of Corti was imaged by confocal microscopy at three positions corresponding to 25%, 50% and 75% of the length of the cochlea measured from the base. The orientation of individual hair cells was measured using the ImageJ (NIH) angle measurement tool. The short arm of each angle was drawn from the pericentrin labeled kinocilium and across the center of the hair cell. The long arm of each angle was drawn parallel to three adjacent hair cells within the same row. This yields a measurement range of 0 to 180 degrees in which a perfectly aligned hair cell has a raw measurement of 90 degrees. For those cells determined to have reversed bundles (i.e. kinocilium pointed toward the spiral ganglia) the orientation was calculated with the formula x = (360-y) where x is orientation and y is the measured angle. This calculation and additional analyses were completed using Microsoft Excel. These absolute measurements of auditory hair cell orientation were assembled as a circular histograms using Oriana circular graphing software (Kovach Computing Services). Planar polarity phenotypes were further quantified by averaging the mean absolute deviation of hair cell polarities from an arbitrary reference (defined as 0°) for each animal as illustrated in Fig. 6B. These analyses were completed for each genotype at the three cochlear positions. Statistical significance was calculated by a two-tailed Student's t-test with unequal variance.

For vestibular hair cell analyses the utricular maculae was imaged by confocal microscopy at two positions spanning the Gata3-positive or Oncomodulin-positive striola and images were combined based upon regions of overlap. As outlined in Fig. 2, stereocilia bundle polarity was measured in three 100 µm×50 µm analysis fields. Using Canvas11 illustration software (Deneba), analysis fields were positioned with field2 centered on the striola. Field1 was positioned in the medial utricle and field3 was in the lateral utricle, with each separated from field2 by 50 µm and 20 µm gaps respectively. The orientation of individual hair cells within each analysis field was marked using the Canvas11 line tool and ROIs were exported to ImageJ for orientation measurements. This yields a measurement range of 0–180°s for cells oriented towards the lateral utricle (i.e. WT cells in field2). For those cells determined to be oriented towards the medial region (i.e. WT cells in field 3) bundle orientation was calculated with the formula x = (180+y) where x is orientation and y is the measured angle. Vestibular hair cell orientation was assembled as a circular histogram using Oriana circular graphing software. Planar polarity phenotypes were further quantified by averaging the mean absolute deviation of hair cell polarities from an arbitrary reference (defined as 0°) for each animal as illustrated in Fig. 3B. Statistical significance was calculated by a two-tailed Student's t-test with unequal variance.

See Table S1 for sample sizes at each position, tissue and genotype. Because there was some variability of severity of NTDs in *vangl2^ΔTMs/ΔTMs^* KOs, only KOs with craniorachischisis were included in measurements of hair cell planar polarity. All *vangl2^ΔTMs/Lp^* and *vangl2^Lp/Lp^* mutants collected had craniorachischisis. Hair cells in which the bundle polarity or pericentrin labeling could not be visualized were not included in the auditory or vestibular analyses.

### MDCK and HEK293 cell culture, electroporation and immunolabeling

MDCK cells (SIGMA 84121903) were grown in EMEM supplemented with 2 mM L-Glutamine, 1× non-essential amino acids, 10% FBS and 1× Penn/Strep. HEK293 cells (American Type Culture Collection ATCC-CRL-1573) were grown in DMEM, 10% FBS and 1× Penn/Strep. MDCK cells were transfected via electroporation, by combining 1×10^7^ cells with 30 ug plasmid DNA (60 ug total for co-electroporations) in a 2 mm gap electroporation cuvette, followed by electroporation at 260 V with 950 uF capacitance and 25 Ohm resistance using a BTX ECM630 electroporation box. MDCK cells were plated into single wells of a Lab-Tek II 8-well Chamber Slide (Thermo Scientific) and incubated 72 hours to allow adherence junctions to form at cell boundaries before the distribution of EGFP-tagged proteins was assayed by immunofluorescent labeling. HEK293 cells were transfected using Lipofectamine reagent as per the manufacturer's recommendation, cultured for 24–48 hours and harvested for Western blot analysis. For these experiments a plasmid expressing EGFP-Vangl1 was generated by PCR amplification from mouse cDNA and cloned into the pEGFP-C1 vector (Invitrogen) to generate an amino-terminus EGFP fusion. EGFP-Vangl2 [7], 3XHA-tagged Vangl2 constructs [27], and E-Cadherin-GFP have been described elsewhere [44]. The E-Cadherin-GFP construct was distributed by Addgene (Addgene plasmid 28009).

For labeling, MDCK cells were fixed with 4% paraformaldehyde prepared in PBS, permeabilized and blocked as described previously. Cells were immunolabeled with anti-HA and anti-GFP antibodies diluted in 1% BSA/PBS followed by Alexa Fluor conjugated secondary antibodies, and then examined by fluorescent microscopy using a Nikon E600 compound microscope. For protein localization assays only cells expressing both HA-tagged and EGFP-tagged constructs were evaluated, and the distribution of EGFP-tagged proteins were scored as membrane associated, cytoplasmic or present in both locations.

### Western blot and Co-immunoprecipitation

Protein lysates were prepared from tissues, MDCK or HEK293 cells using a lysis buffer consisting of 25 mM Tris pH 7.4, 1% Triton X-100, 50 mM NaCl and 1× protease inhibitor cocktail (SIGMA P8340) and quantified by UV absorption at 280 nm using a NanoDrop. Following SDS-PAGE electrophoresis proteins were transferred to nitrocellulose filters and blotted with standard Western blot techniques using anti-GFP or anti-HA antibodies followed by chemiluminescent detection using BioRad Immun-Star HRP substrate. Immunoprecipitations were completed using Nynal protein G beads (Invitrogen) bound to rabbit anti-GFP or mouse anti-HA antibodies and combined with protein lysates from electroporated MDCK cells. Prior to immunoprecipitation, levels of recombinant protein expression were determined by Western blot analysis and equivalent amounts of recombinant protein were used from each electroporation condition. Target antigens were absorbed at 4°C, and washed using the manufacturers recommended protocols and solutions, then eluted from the beads using Laemmli 2× Gel-loading Buffer with DTT prior to denaturing SDS-PAGE and conventional Western blot analysis.

## Supporting Information

Figure S1 **Gata3 and Oncomodulin immunolabeling marks the position of the striola in developing mouse utricle.** (**A**) The transcription factor Gata3 (red) is expressed by multiple cells types located in the striola region of the developing utricle and Gata3 immunolabeling can be used to visualize this region. (**B**) The calcium binding protein Oncomodulin (red) is expressed exclusively by type1 hair cells located in the striola, and Oncomodulin immunolabeling can also be used to visualize this region. (**A–B**) Phalloidin (green) was used to assay stereocilia bundle orientation (arrows) and map the position of the line of polarity reversal (LPR, dashed lines). In mouse, the LPR is located along the lateral border of the striola.
(TIF)

Figure S2 **Ectopic expression of tagged-Vangl1 or Vangl2 proteins does not disrupt cell junctions between adjacent MDCK cells.** (**A–E**) The proper formation of adherens junctions between adjacent MDCK cells was determined by immunofluorescent labeling of endogenous E-cadherin (red) following electroporation with EGFP-Vangl1 (A, green), EGFP-Vangl2 (B, green), 3XHA-Vangl2 (C, green), 3XHA Vangl2^D255E^ (D, green) or 3XHA Vangl2^S464N^ (E, green) constructs. Asters mark transfected cells. Consistent with experiments utilizing E-Cadherin-EGFP (Fig. 7M–R), transgenic expression of Vangl1/2 proteins does not disrupt E-Cadherin distribution at cellular junctions.
(TIF)

Figure S3 **Specificity of anti-Vangl1 antibody evaluated by Western blot.** (**A**) Western blot against protein lysates from embryonic tissues demonstrate that the anti-Vangl1 antibody does not cross react with endogenous Vangl2 protein. Blots show a prominent band at the predicted 70 Kd mass in Wt and *vangl2^ΔTMs/WT^* lysates and the labeling pattern is not altered in *vangl2^ΔTMs/ΔTMs^* mutant tissue. Blots for Tubulin were used as a loading control. (**B**) Western blot against cell lysates from HEK293 cells transfected with EGFP-Vangl1 or EGFP-Vangl2 reveal that the anti-Vangl1 antibody can cross-react with recombinant Vangl2 protein when over-expressed. Serial dilutions of transfected cell lysates show the relative affinity of the anti-Vangl1 antibody and demonstrate that it can detect a 10-fold lower concentration of EGFP-Vangl1 than EGFP-Vangl2. The amount of total protein in each lysate was determined by UV absorption measured with a NanoDrop and equivalent amounts were loaded into each lane. The amount of total protein lysate per lane is indicated.
(TIF)

Table S1 **Sample size summary for planar polarity phenotypic analysis.** The number of experimental and control animals used for quantification of the averaged mean deviation of bundle orientations in vestibular assays and auditory assays completed at each of the three positions along the length of the cochlea. Different numbers of specimen were available at each location because some were excluded based upon dissection or labeling artifacts.
(PDF)
