# Correction: Comparison of Phenotypes between Different *vangl2* Mutants Demonstrates Dominant Effects of the *Looptail* Mutation during Hair Cell Development

**DOI:** 10.1371/annotation/b5a73429-7f31-4c89-b93e-4e0a08eb4dd4

**Published:** 2012-05-31

**Authors:** Haifeng Yin, Catherine O. Copley, Lisa V. Goodrich, Michael R. Deans

There is an error in affiliation 2 for author Lisa V. Goodrich. Affiliation 2 should be: The Department of Neurobiology, Harvard Medical School, Boston, Massachusetts, United States of America.

