# Supplementary figures and images for: Comparison of Phenotypes between Different vangl2 Mutants Demonstrates Dominant Effects of the Looptail Mutation during Hair Cell Development
Source: PLoS One. 2012 Feb 20;7(2):e31988. doi: 10.1371/journal.pone.0031988 (PMC3282788; doi:10.1371/journal.pone.0031988)

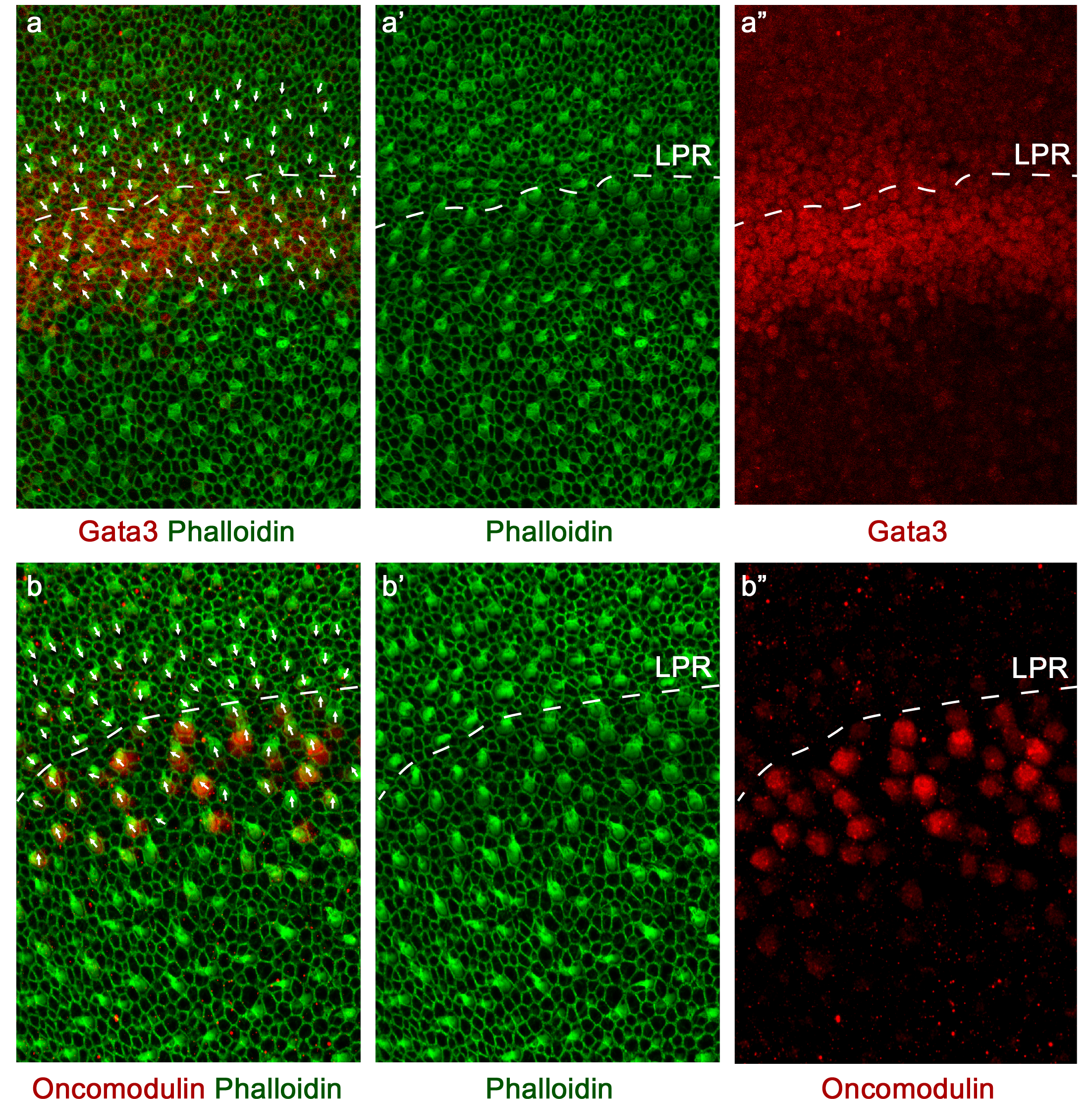

Supplement: Figure S1 — Gata3 and Oncomodulin immunolabeling marks the position of the striola in developing mouse utricle. (A) The transcription factor Gata3 (red) is expressed by multiple cells types located in the striola region of the developing utricle and Gata3 immunolabeling can be used to visualize this region. (B) The calcium binding protein Oncomodulin (red) is expressed exclusively by type1 hair cells located in the striola, and Oncomodulin immunolabeling can also be used to visualize this region. (A–B) Phalloidin (green) was used to assay stereocilia bundle orientation (arrows) and map the position of the line of polarity reversal (LPR, dashed lines). In mouse, the LPR is located along the lateral border of the striola. (TIF) [file pone.0031988.s001.tif]

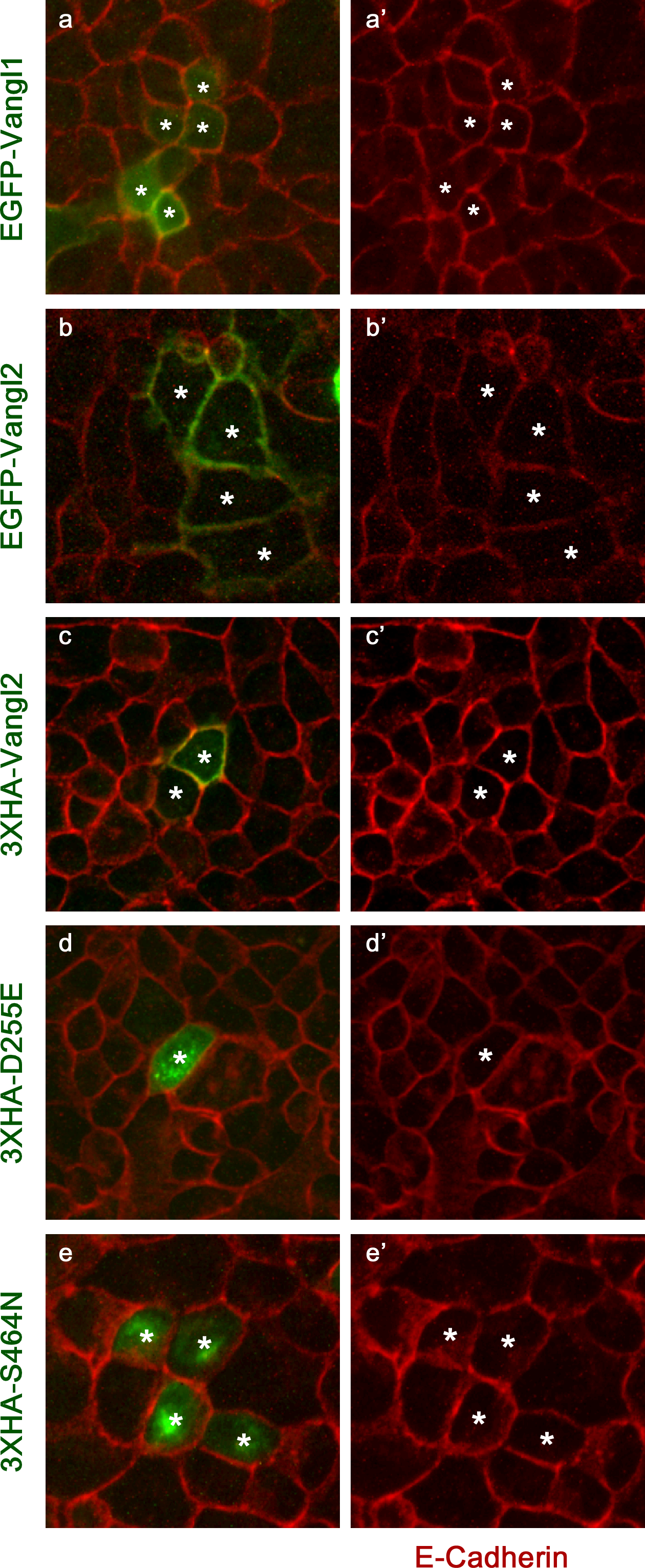

Supplement: Figure S2 — Ectopic expression of tagged-Vangl1 or Vangl2 proteins does not disrupt cell junctions between adjacent MDCK cells. (A–E) The proper formation of adherens junctions between adjacent MDCK cells was determined by immunofluorescent labeling of endogenous E-cadherin (red) following electroporation with EGFP-Vangl1 (A, green), EGFP-Vangl2 (B, green), 3XHA-Vangl2 (C, green), 3XHA Vangl2D255E (D, green) or 3XHA Vangl2S464N (E, green) constructs. Asters mark transfected cells. Consistent with experiments utilizing E-Cadherin-EGFP (Fig. 7M–R), transgenic expression of Vangl1/2 proteins does not disrupt E-Cadherin distribution at cellular junctions. (TIF) [file pone.0031988.s002.tif]

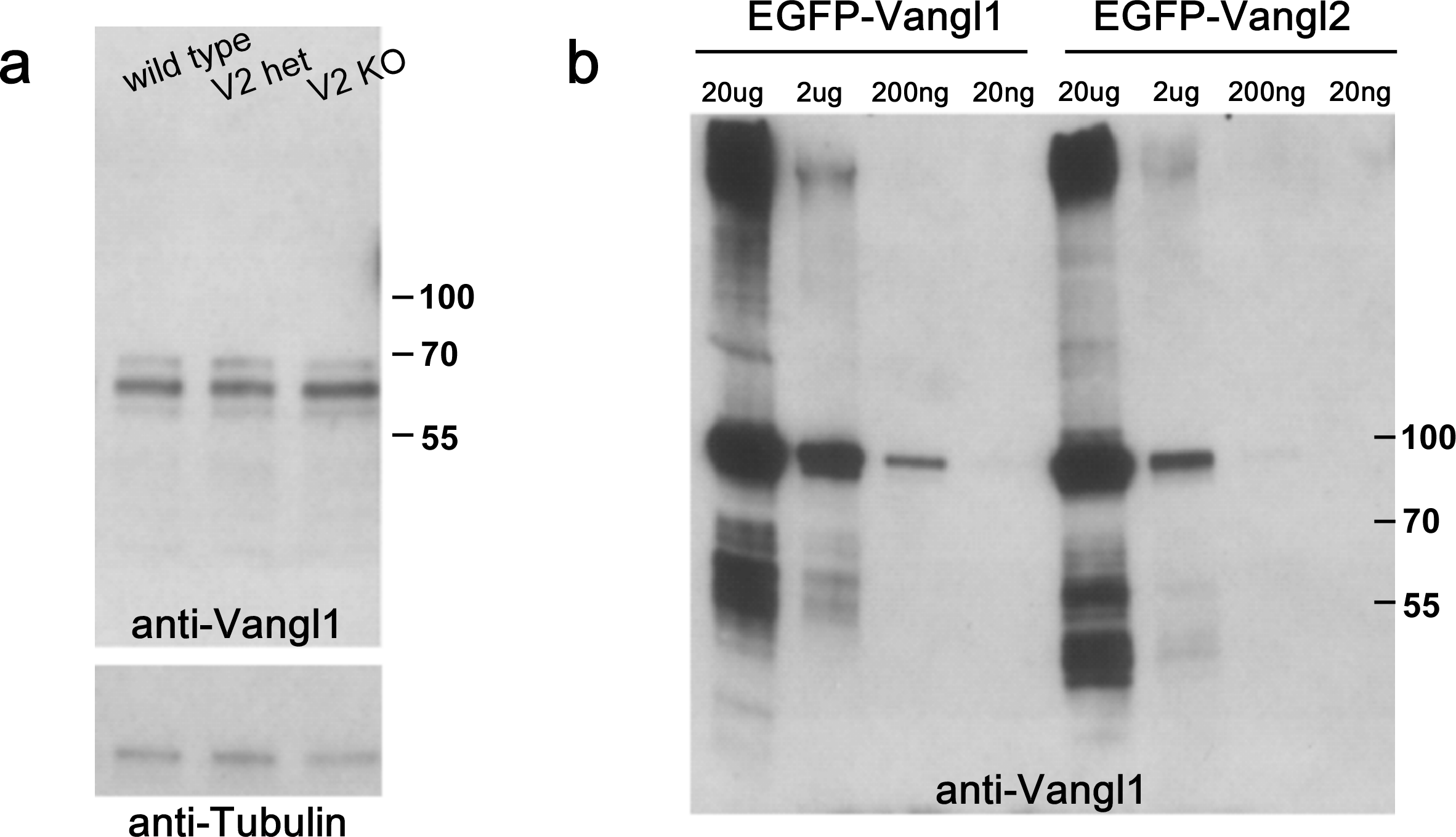

Supplement: Figure S3 — Specificity of anti-Vangl1 antibody evaluated by Western blot. (A) Western blot against protein lysates from embryonic tissues demonstrate that the anti-Vangl1 antibody does not cross react with endogenous Vangl2 protein. Blots show a prominent band at the predicted 70 Kd mass in Wt and vangl2ΔTMs/WT lysates and the labeling pattern is not altered in vangl2ΔTMs/ΔTMs mutant tissue. Blots for Tubulin were used as a loading control. (B) Western blot against cell lysates from HEK293 cells transfected with EGFP-Vangl1 or EGFP-Vangl2 reveal that the anti-Vangl1 antibody can cross-react with recombinant Vangl2 protein when over-expressed. Serial dilutions of transfected cell lysates show the relative affinity of the anti-Vangl1 antibody and demonstrate that it can detect a 10-fold lower concentration of EGFP-Vangl1 than EGFP-Vangl2. The amount of total protein in each lysate was determined by UV absorption measured with a NanoDrop and equivalent amounts were loaded into each lane. The amount of total protein lysate per lane is indicated. (TIF) [file pone.0031988.s003.tif]
