# Supplementary material for: Comparison of Phenotypes between Different vangl2 Mutants Demonstrates Dominant Effects of the Looptail Mutation during Hair Cell Development
Source: PLoS One. 2012 Feb 20;7(2):e31988. doi: 10.1371/journal.pone.0031988 (PMC3282788; doi:10.1371/journal.pone.0031988)
Supplement: Table S1 — Sample size summary for planar polarity phenotypic analysis. The number of experimental and control animals used for quantification of the averaged mean deviation of bundle orientations in vestibular assays and auditory assays completed at each of the three positions along the length of the cochlea. Different numbers of specimen were available at each location because some were excluded based upon dissection or labeling artifacts. (PDF) [file pone.0031988.s004.pdf]

Genotype	Utricular Analyses				Cochlear Analyses					
		field 1	field 2	field 3	25% Cochlear length		50% Cochlear length		75% Cochlear length	
	# of mice	# of hair cells			# of mice	# of hair cells	# of mice	# of hair cells	# of mice	# of hair cells
**Wild Type**	n=10	n=317	n=295	n=291	n=6	n=607	n=5	n=587	n=3	n=290
vangl2 $^{\Delta TMs/WT}$	n=6	n=182	n=204	n=182	n=4	n=426	n=4	n=419	n=4	n=346
vangl2 $^{Lp/WT}$	n=2	n=60	n=46	n=51	n=3	n=269	n=3	n=259	n=3	n=260
vangl2 $^{\Delta TMs/\Delta TMs}$	n=5	n=144	n=140	n=115	n=6	n=545	n=5	n=476	n=4	n=367
vangl2 $^{\Delta TMs/Lp}$	n=4	n=108	n=128	n=102	n=5	n=479	n=3	n=299	n=3	n=285
